# Editorial: Integrating computational modeling and organoid technology for enhanced biological research

**DOI:** 10.3389/fbioe.2025.1670630

**Published:** 2025-09-19

**Authors:** J. M. García-Aznar, E. McEvoy, S. W. Verbruggen, P. Van Liedekerke

**Affiliations:** ^**1**^ Aragon Institute of Engineering Research (I3A), Multiscale in Mechanical and Biological Engineering (M2BE), Universidad de Zaragoza, Zaragoza, Spain; ^2^ School of Engineering and Architecture (EINA), Department of Mechanical Engineering, Universidad de Zaragoza, Zaragoza, Spain; ^3^ Biomedical Engineering, College of Science and Engineering, University of Galway, Galway, Ireland; ^4^ CÚRAM, Research Ireland Centre for Medical Devices, University of Galway, Galway, Ireland; ^5^ Centre for Bioengineering and Centre for Predictive in vitro Models, School of Engineering and Materials Science, Queen Mary University of London, London, United Kingdom; ^6^ Department of Data Analysis and Mathematical modeling, Ghent University, Ghent, Belgium

**Keywords:** organoids, in vitro models, computational models, mathematical tools, Bayesian calibration methods, numerical simulations

Organoids are self-organized 3D cell-based *in vitro* models that replicate the key functional, structural and biological complexities of organs (Zhao et al., 2022). Depending on the application, organoids can be derived from either pluripotent or tissue-resident stem (embryonic or adult) or progenitor or differentiated cells from healthy or diseased tissues, such as tumors (Zhao et al., 2022; Kim et al., 2020). Therefore, organoid technology is a novel approach to study pathologies and their treatment, providing a personalized strategy. Despite their potential applicability, organoids exhibit a high level of complexity that requires advanced mathematical and computational models for comprehensive understanding, being mathematical and computational models an adequate strategy as it has been shown in recent works (Camacho-Gomez et al., 2023; McEvoy et al., 2020; Van Liedekerke et al., 2019).

Since organoids are relatively small systems with a potentially high spatial variability in both biophysical parameters as well as genetic parameters, we can advocate the use of spatio-temporal models such as continuum-based approaches (McEvoy et al., 2020) and agent-based approaches (Van Liedekerke et al., 2019), allowing a description of the local cell-specific biophysical variables in space and time. Agent-based models are a bottom-up approach that allows for simulations of emergent behavior in multi-cellular systems from extensive cell-cell interactions. These include intracellular models that describe the cell state and decision mechanisms for each individual cell, and are typically highly-dimensional with regard to the chemical species and their reactions involved. To alleviate the high complexity and computational burdens, concepts from machine learning techniques can be introduced. For example, in (Camacho-Gomez et al., 2023), a hybrid agent-based approach with a trained neural network as intracellular state decision model was proposed.

However, despite such advances there remains a gap in validating and integrating these computational tools with experimental research to achieve a more quantitative and predictive understanding of organoid dynamics and physiology. Combining numerical simulations results with experimental data requires rigorous model verification, calibration and validation. Mathematical tools such as global sensitivity analysis, Bayesian calibration methods and cross-validation methods can provide a path to more consistent model development (Lima et al., 2021; Hervas-Raluy et al., 2023).

The goal of leveraging various numerical and mathematical approaches is to advance the understanding of organoid technology, from their morphogenesis and development to their functionality. This Editorial contributes to how numerical tools can improve our understanding of *in vitro* experiments. We believe that this Research Topic will lead to new strategies and methodologies for understanding the role and functionality of organoids as well as a more rapid utilization in medicine. Yet, specific research questions still need to be addressed. Organoids are systems with a high complexity and variety, entailing processes operating at different scales and requiring different state-of-the-art modelling techniques. Which different modeling techniques are most suitable to characterise a specific organoid, and which experimental data are most informative for a given option? It is becoming increasingly clear that hybrid mechanistic data-driven approaches of techniques (such as mentioned before) represent a promising strategy.
